# Climatology of severe hail potential in Europe based on a convection-permitting simulation

**DOI:** 10.1007/s00382-024-07227-w

**Published:** 2024-05-18

**Authors:** Abdullah Kahraman, Elizabeth J. Kendon, Hayley J. Fowler

**Affiliations:** 1 School of Engineering, Newcastle University, Newcastle upon Tyne, UK; 2 Met Office Hadley Centre, Exeter, UK; 3 Visiting Scientist at Met Office Hadley Centre, Exeter, UK

**Keywords:** Hail, Hailstorms, Severe thunderstorms, Convection-permitting simulations, Severe weather, Europe

## Abstract

We present a new approach to identify severe hailstorms in a convection-permitting climate model, and build a climatology of severe hail potential in Europe using an ingredients-based approach based on a 20-year long hindcast simulation. Severe hail in Europe occurs mostly in southern regions (up to 40 times a year per 10,000 km^2^ around Northern Italy), and from May to August. It peaks from afternoon to evening hours on land, whilst sea areas are prone to hail at any time of the day. The Mediterranean Sea experiences severe hailstorms mostly in autumn: the central Mediterranean has the highest frequency among all regions studied, and may be considered as an unknown alley for hailstorms in Europe. Results derived from the high-resolution model are in very good agreement with existing hail climatologies constructed from observations, including the fine scale spatial variation. We conclude that our approach provides a reliable proxy for studying future changes in severe hail in convection-permitting simulations.

## Introduction

Hail is a convective weather hazard that is relatively rare at individual locations, but results in significant impacts including damage to property, even sometimes direct fatalities (Allen et al. 2020; Cerveny et al. 2017). It can damage crops, cars, buildings, and animals, especially when it is large and occurs alongside severe winds such as strong downdrafts from thunderstorms. Planes also are affected by hailstorms. Melting hail and blockages from large hail accumulations can also lead to or occur along with flash floods (e.g., Grahame et al. 2009; Chapell and Rodgers 1988), which can halt traffic and cause fatalities.

Since hail is a highly localized meteorological event, with swaths typically a few kilometres wide and one order of magnitude longer in length (the most severe ones are up to a few hundred kilometres), lasting generally only for a few minutes in a given locality, only a small fraction of hailstorms are recorded by meteorological station networks (Allen and Tippett 2015). Historically, those that we know of are generally major hailstorms, resulting in damage to property, or directly witnessed by the people who record them (Kahraman et al. 2016). Hailstorms that occur in rural areas with low population density, or mountains, forests, deserts, or over ocean and sea areas are rarely reported, as are those that occur during night-time (Allen and Tippett 2015). Nevertheless, it is possible to detect storms that likely produce hail via remote sensing tools such as meteorological radars (especially dual-polarisation radars; Kumjian 2013) and satellite products (Bedka et al. 2018). However, the spatial coverage of remotely-sensed products is not homogeneous, and these do not assess whether hail reaches the ground, alongside other limitations (Kunz and Puskeiler 2010; Ortega 2018).

Previous studies of the spatial, temporal, and intensity distributions of hail in Europe mostly rely on national or local datasets (e.g., Tuovinen et al. 2009; Nisi et al. 2018; Sioutas et al. 2009) collected by hydrometeorological services or institutions. Since the European country sizes are relatively small, as well as very few localities with hailpad networks (Manzato 2012; Fraile et al. 1999; Merino et al. 2014), these studies provide an incomplete picture of European hail climatology, although their essential insights are very valuable.

The recent decade has seen a rising interest in a pan-European approach to hail research. Climatologies based on observations / severe weather reports (Púčik et al. 2019; Taszarek et al. 2020a; Hulton and Schultz 2023), the synthesis of country-based climatologies (Punge and Kunz 2016), remote sensing tools combined with other forms of data (Punge et al. 2017), reanalyses (Rädler et al. 2018; Taszarek et al. 2020b) and climate models with parameterized convection (Mohr et al. 2015; Rädler et al. 2019) have been used to provide a holistic view of hail hazard across Europe. Generally, observational studies suffer from incomplete datasets due to under-reporting issues (as discussed in Taszarek et al. 2020a), while model-based studies rely on proxies that approximate the environmental conditions of hail, and are sensitive to model biases. In short, there is no perfect hail climatology, with different approaches having different strengths and weaknesses.

Climate modelling has undergone a paradigm shift in the recent decade, with an increase in the resolution of projections to kilometre-scale (Kendon et al. 2014, 2023; Coppola et al. 2020; Ban et al. 2021). Long climate simulations at convection-permitting resolution are now possible, giving an improved representation of convective storms (Prein et al. 2015). These simulations explicitly represent the features of hailstorms, including updrafts, downdrafts, and the formation of ice particles. They add value especially in terms of storm dynamics, local wind systems, and the interaction of atmospheric flows with topography (Prein et al. 2015; Houze 2012). It is possible to produce a multi-decadal climatology of hail without relying on environmental proxies. This study is the first to exploit a CPM to produce a hail climatology for Europe. We use a Met Office Unified Model simulation with 2.2 km horizontal grid spacing, spanning 20 years. We describe the data, modelling aspects and the novel hail proxy developed here in Sect. 2. In Sect. 3, we present the resulting climatology and its comparison to existing climatologies. Finally, Sect. 4 concludes the paper.

## Data and methods

### The hindcast simulation

**Fig. 1:**
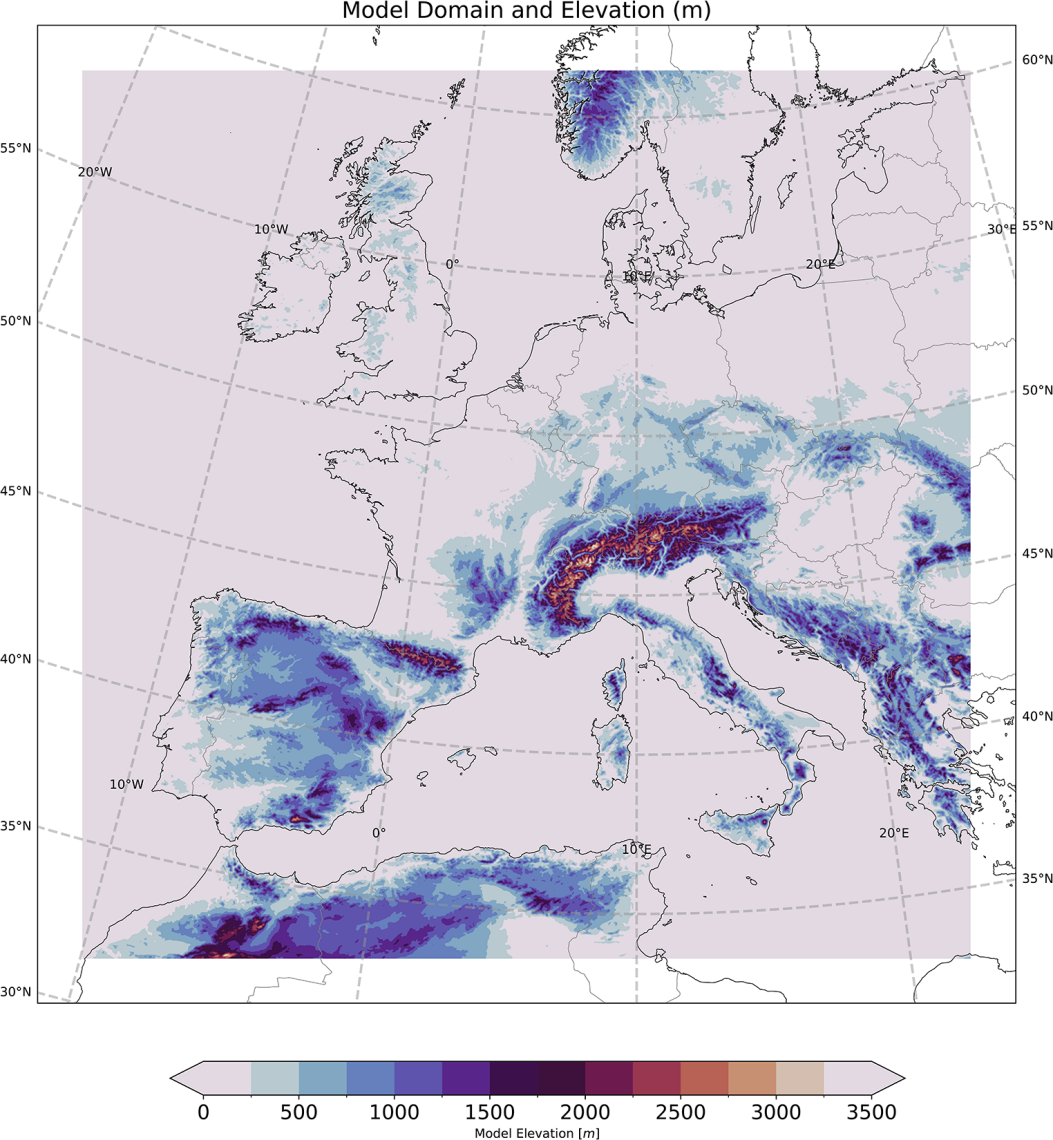
Full domain (black frame), and the analysis region, excluding 70 grid cells from each boundary (terrain coloured by model elevation, in m)

A pan-European convection-permitting simulation has been conducted using the Met Office Unified Model v10.1, using a configuration based on the operational forecasting model. The simulation uses ERA-Interim (Dee et al. 2011) to provide initial and boundary conditions (every 6 h), without nudging, providing a downscaled representation of a historical period, termed a “hindcast” (ERA-Interim was used since ERA5 was not available at the time when the simulation was run). This simulation is not a reanalysis replacement, but part of a climate change experiment, so the configuration is set accordingly as the eventual aim is to investigate future changes. The rotated pole (located at 43°N, 190°E) domain covers most of Europe, with 1536 × 1536 horizontal grid cells, and ∼ 2.2 km spacing. Due to the large resolution difference between ERA-Interim (0.75°) and the downscaled regional model, we exclude 70 grid points from each boundary (∼ 154 km) prior to our analysis to allow for the spin-up of small-scale features. The full domain and the topography of the analysis region are shown in Fig. 1.

The Unified Model’s dynamics, “Even Newer Dynamics for General atmospheric modelling of the environment” (ENDGame), is based on a semi-implicit semi-Lagrangian discretization of the governing equations (Wood et al. 2014). It is fully compressible and nonhydrostatic. The model uses Arakawa-C grid staggering in the horizontal. Terrain-following hybrid height coordinates are used in the vertical. The model top is set to 40 km, and the lowest vertical level is 2.5 m above the surface. There are 70 vertical levels, with spacing increasing quadratically with height. The model time step is set to 60 s.

As convection is assumed to be resolved by the model dynamics at such km-scale resolutions, no convection parameterization is used. We acknowledge that small-scale convection is not fully resolved [resulting in updrafts being too large with too little entrainment (e.g., Wang et al. 2020; Prein et al. 2021)], but for the purposes of severe hailstorm research, our focus is on the larger storms that are better resolved. Radiation parameterization is based on Edwards and Slingo (1996), with upgrades described in Stratton et al. (2018). The radiative time step is 15 min, with 5-min “correction” updates. A modified Wilson and Ballard microphysics scheme (Wilson and Ballard 1999; Williams et al. 2018) is used, capturing graupel, water vapour, cloud liquid, cloud ice and rain. It is a single-moment scheme, using physics-based transfer equations to predict ice (and graupel, separately), along with liquid water content as prognostic variables. The transfers include condensation and evaporation between water vapour and liquid, riming (from liquid into ice), evaporation from rain, nucleation as well as deposition/sublimation between ice and vapour phases, Bergeron process, accretion/auto-conversion (from liquid droplets to rain), and ice/graupel/rain conversions (melting, capture etc) falling in and out. Equations are provided in the Appendices of Wilson and Ballard (1999). The boundary layer scheme has been developed for high resolution simulations, as described by Boutle et al. (2014). The land surface model used is the Joint UK Land Environment Simulator (JULES, Best et al. 2011; Clark et al. 2011).

Further details about the model and its performance in simulating precipitation extremes is presented in Berthou et al. (2020). In summary, they find that the 2.2 km model leads to reduced biases compared to a 12 km model with parametrised convection, with an improved diurnal cycle of precipitation and more intense extremes, and a better representation of Mediterranean autumnal intense events with more realistic mesoscale processes.

The simulation spans 21 years, the first of which is discarded as model spin-up. All analysis is performed on 20 years of data, from 1 March 1999 to 28 February 2019. Most of the analysis is based on instantaneous data, at 3-hour intervals.

### The hail proxy

#### Background: hail formation theory in brief

Hail requires three ingredients. The first is an embryo, such as graupel, small frozen drops, or sometimes other particles, which make up the nuclei of hailstones (Knight and Knight 1970). The second is an abundance of supercooled water droplets, which can be collected by the embryo for further growth, via accretion or riming processes. Hail growth occurs at temperatures between − 10 and − 30 °C (e.g., Knight et al. 1975; Ziegler et al. 1983), as either “dry” or “wet” growth regimes (or a combination of both), based on available liquid water, ambient temperature, hailstone surface temperature, and its size. This layer is known as the hail growth zone (HGZ). The final ingredient is time for these processes to occur: typically 10 minutes or longer for growth to “large” sizes (Allen et al. 2020). “Large” is usually defined as a maximum diameter of 2 cm in Europe and 1” in the United States (previously 0.75”); this is considered as “severe” hail.

Supercells are known to produce the largest hailstones, and the growth mechanism within them is well-known (e.g., Morgan 1972; Browning and Foote 1976). A rotating updraft, called a mesocyclone, is the unique feature of a supercell. Hail embryos may be formed in flanking line convection or grown entirely within the main updraft, more likely at its weaker periphery (Heymsfield and Musil 1982). The embryos injected into the updraft via environmental storm-relative flow grow rapidly in the liquid-rich updraft, following a path of ascent and then descent. The amount of time spent in the HGZ is determined by the balance between the hailstone fall speed and the updraft speed (Browning 1964, among many others, e.g., Morgan 1972; Heymsfield 1983; Miller and Fankhauser 1983; Foote 1984; Musil et al. 1986). If the fall speed is larger, the particle falls quickly. Conversely, if the updraft speed is larger it is ejected out of the growth zone. A balance is necessary to keep the particle suspended within the hail growth layer, with only horizontal movement within the updraft, sweeping up supercooled water droplets. For this balance to be maintained during the growth of the hailstone, the particle should be moving towards the stronger parts of the updraft with time, i.e. the trajectory of the hailstone should follow a path from the periphery of the updraft towards the centre. These conditions are best provided by mesocyclones, especially wider (and stronger) ones.

#### Extracting hail environments from model data

Hail ingredients and processes occur at small scales, and so are typically poorly represented in climate models. Instability indices and the convective available potential energy (CAPE) typically used as proxies within traditional climate models indirectly provide information on possible updraft speeds, but have been found to be inadequate for hail estimation (e.g., Doswell 1982; Groenemeijer and van Delden 2007; Mohr and Kunz 2013). In a km-scale model, we have the possibility to directly extract the updraft speeds favourable to hail formation as described above. We focus on severe hail because such storms are more easily discerned from other thunderstorms, and because these suffer less from under-reporting issues in observations allowing more reliable verification.

When a parameter related to updraft strength is combined with another one associated with kinematic environments, such as wind shear or storm-relative environmental helicity, it is found to better represent large (or severe) hail environments (e.g. Johnson and Sugden 2014; Kahraman et al. 2017), as these kinematic environments are the crucial determinants of thunderstorm organization (e.g., larger deep layer shear or storm relative helicity favours supercells, ideal settings for hail growth) and wider mid-level updrafts (Trapp et al. 2017; Dennis and Kumjian 2017). Recent studies have shown that high wind shear at low levels is unfavourable for hail production since hailstones are then rapidly advected out of the hail growth region, and that straighter hodographs (diagrams that depict the vertical distribution of horizontal winds) support larger hail production (Kumjian and Lombardo 2020; Kumjian et al. 2021).

Freezing level height is commonly used to identify environments favourable for severe hail (e.g., Billet et al. 1997; Kitzmiller and Breidenbach 1993; Edwards and Thompson 1998; Prein and Holland 2018), as it helps to distinguish too warm or too dry environments, which might be unfavourable for hail growth. It is also important for the melting of hail: a higher freezing level results in more melting of hailstones (especially smaller ones) before they reach the ground (e.g., Brimelow et al. 2017).

Other predictors include lapse rates, moisture contents, different versions of wind parameters, and composite parameters such as the Significant Hail Parameter (SPC 2014) or Large Hail Parameter (Johnson and Sugden 2014). Usually, such parameters are found to be most useful for the region in which they have been developed (mainly the Great Plains of the United States), due to unique local climatological features (such as westerly dry air aloft, coming from the Rocky Mountains, and southerly moist advection from the Gulf Of Mexico at low levels), and perform poorly when applied elsewhere (Allen et al. 2020). This is in part due to the choice of layers, which does not represent the intended parcels functioning in different geographical regions.

#### Hail proxies in CPM simulations

A convection-permitting model produces more realistic thunderstorm features compared to coarse resolution models, as well as a more detailed view of their environments. It is possible to exploit simulated updrafts individually for potential hailstorms. For instance, coupled hail models like HAILCAST (Brimelow et al. 2002) performed better in convection-permitting simulations, albeit with further modifications (Adams-Selin and Ziegler 2016).

We build our proxy using parameters relevant to severe hail derived from the available raw model output: graupel (graupel water path 100 or higher), updrafts in the HGZ (WHGZ, 10 m s^− 1^ or higher), vertical wind shear between the lower troposphere and 500 hPa level (DLS, 10 m s^− 1^ or higher), and the freezing level height (FZLV, between 2 and 4.5 km above surface). Grid cells with HGZ updrafts reaching at least 10 m s^− 1^ (where graupel exists) are candidates for severe hail production. We use a relatively low threshold since the data we use are instantaneous snapshots (3-hourly frequency), and it is likely that updrafts are stronger between snapshots. The DLS is calculated as the bulk shear between the lowest available pressure level (from 925 hPa, 850 hPa, and 700 hPa) and 500 hPa. Here, we purposely do not use 10 m wind components, as these are contaminated by realised convection in the model. The most recent research (Kumjian and Lombardo 2020; Kumjian et al. 2021) suggest using a shear layer from approximately 1 km above the surface to the mid-troposphere (rather than the traditional 0–6 km layer comparable to our choice of wind shear layer. We set the shear threshold to 10 m s^− 1^ as this is considered a discriminatory value for severe / non-severe thunderstorms. Finally, the FZLV criteria is set at 2000 m and 4500 m, similar to the range found by Prein and Holland (2018), for example.

**Fig. 2:**
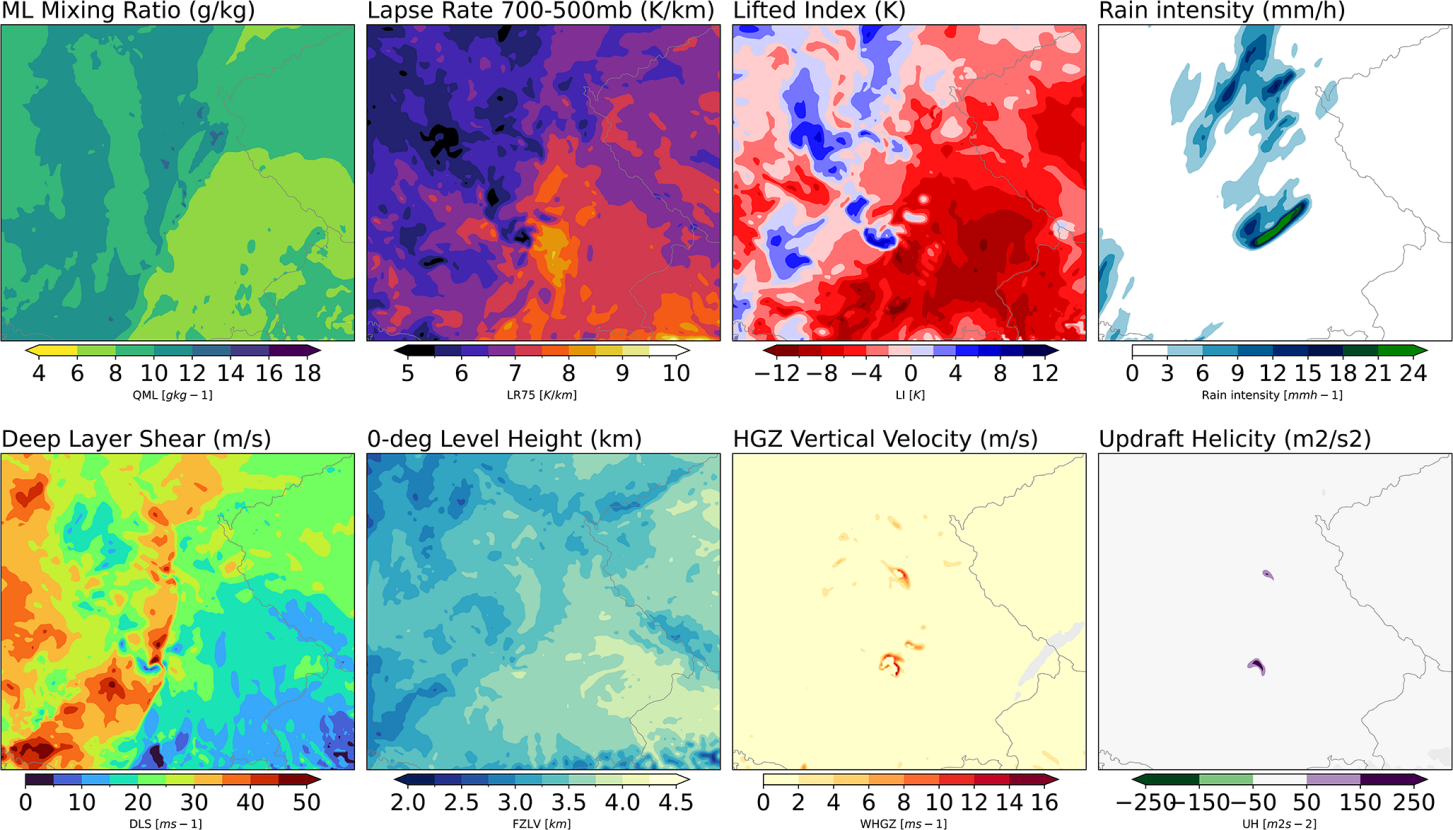
Visualisation of a set of hail-relevant parameters in model simulation, with an instantaneous snapshot from 8 July 2004. Plots show mixing layer mixing ratio, 700 − 500 mb lapse rate, Lifted Index, rain intensity, deep layer shear, 0-degree level height, HGZ vertical velocity–(where graupel exists), and updraft helicity during a prospective severe hail event in Southeastern Germany in the model output

Figure 2 shows a selection of hail-relevant fields for an example of a potential severe hail event identified in the model output. A band of low-level moist air (with mixed-layer mixing ratio values reaching ∼ 15 g kg^− 1^, and locally high lapse rate values in the 700 − 500 hPa layer (9 K km^− 1^), set the scene for high latent instability (lifted index ∼-10 K). The convection-permitting model initiates storms in these regions and produces precipitation. High environmental wind shear is present (15 ∼ 25 m s^− 1^ to the east of the moisture gradient, and much higher in the west), and the 0-degree level height is ∼ 3 to ∼ 4 km above ground, which is adequate for severe hail based on the literature (e.g., Prein and Holland 2018). Vertical velocities of 10 m s^− 1^ in the hail growth zone (-10 °C to -30 °C layer) are reached, where graupel is produced. Furthermore, two of the storms feature high updraft helicity values, indicating right-moving supercells.

**Fig. 3:**
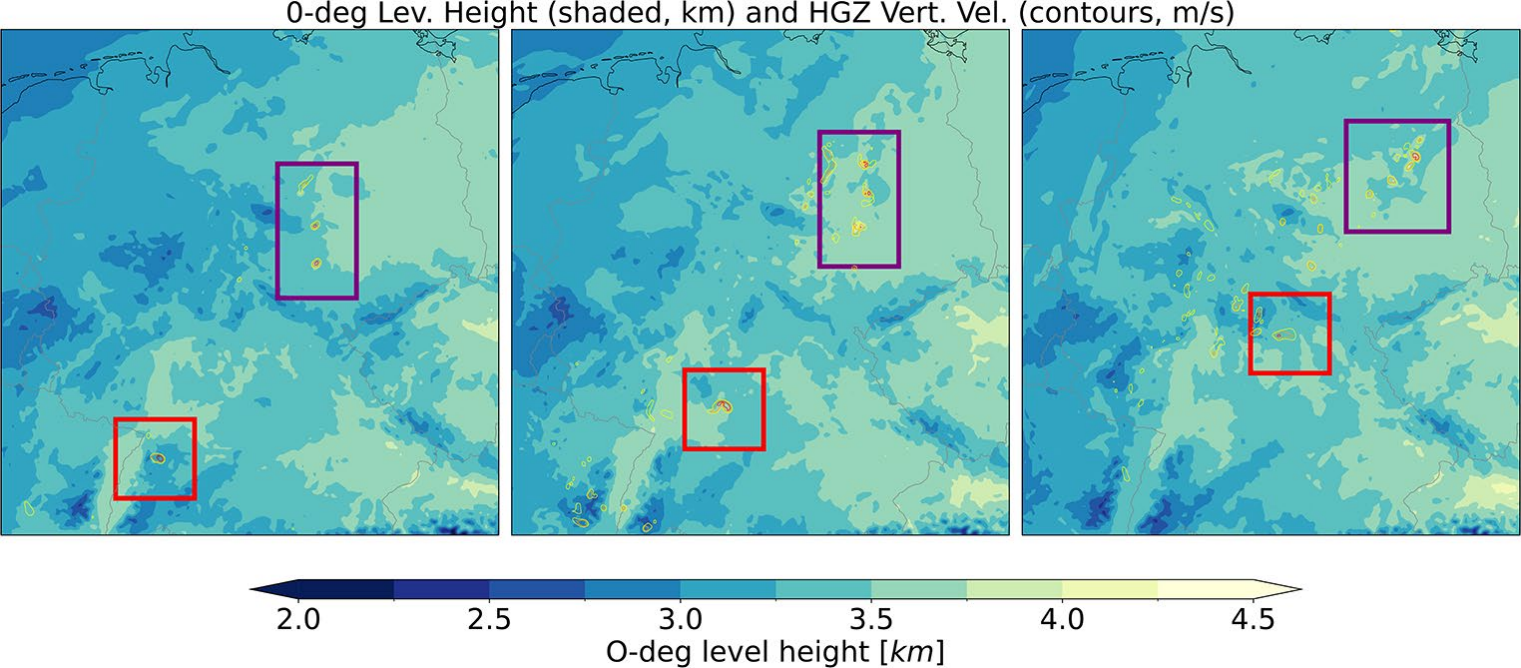
Hail growth zone vertical velocity (where graupel exists) at successive 3-hour intervals, during a severe hail event over Germany in the 2.2 km model (9 June 2010). Yellow contours are for 1 m s^− 1^, orange for 3 m s^− 1^, and red for 10 m s^− 1^ (the threshold for severe hail potential). 0-degree level height is plotted as shaded colour background

Another useful example to demonstrate the evolution of storms within the model is shown in Fig. 3. Here, strong updrafts in the hail growth zone (where graupel exists) are visualized at 3-hour intervals. With a south-westerly background flow, it is possible that the storms highlighted in the red and purple boxes are the same set of storms sampled at different times, because some supercells live up to 6 h (and even longer). Alternatively, short-lived storms may disappear in the “blind” period between model output times and be replaced by newly initiated storms. There is no way to know with only 3-hourly instantaneous output. The severe hail potential we extract here is therefore not equal to the number of hailstorms, or severe hail cases, in the model, but in a 20-year hindcast we assume that the overall geographical distribution of such events represents the hail climatology in a broad sense.

Conventionally, a measure of instability combined with vertical wind shear has been considered a basic tool for assessing severe hail environments (as discussed in Sect. 2.2.2). In our case, as we can extract the simulated updraft within a convection-permitting model, instead of the instability parameters, we use the vertical velocity itself (along with the shear), to look for the potential for hail. For instance, higher values of the multiplication of vertical velocity in the hail growth zone and wind shear could favour even larger hail.

**Fig. 4:**
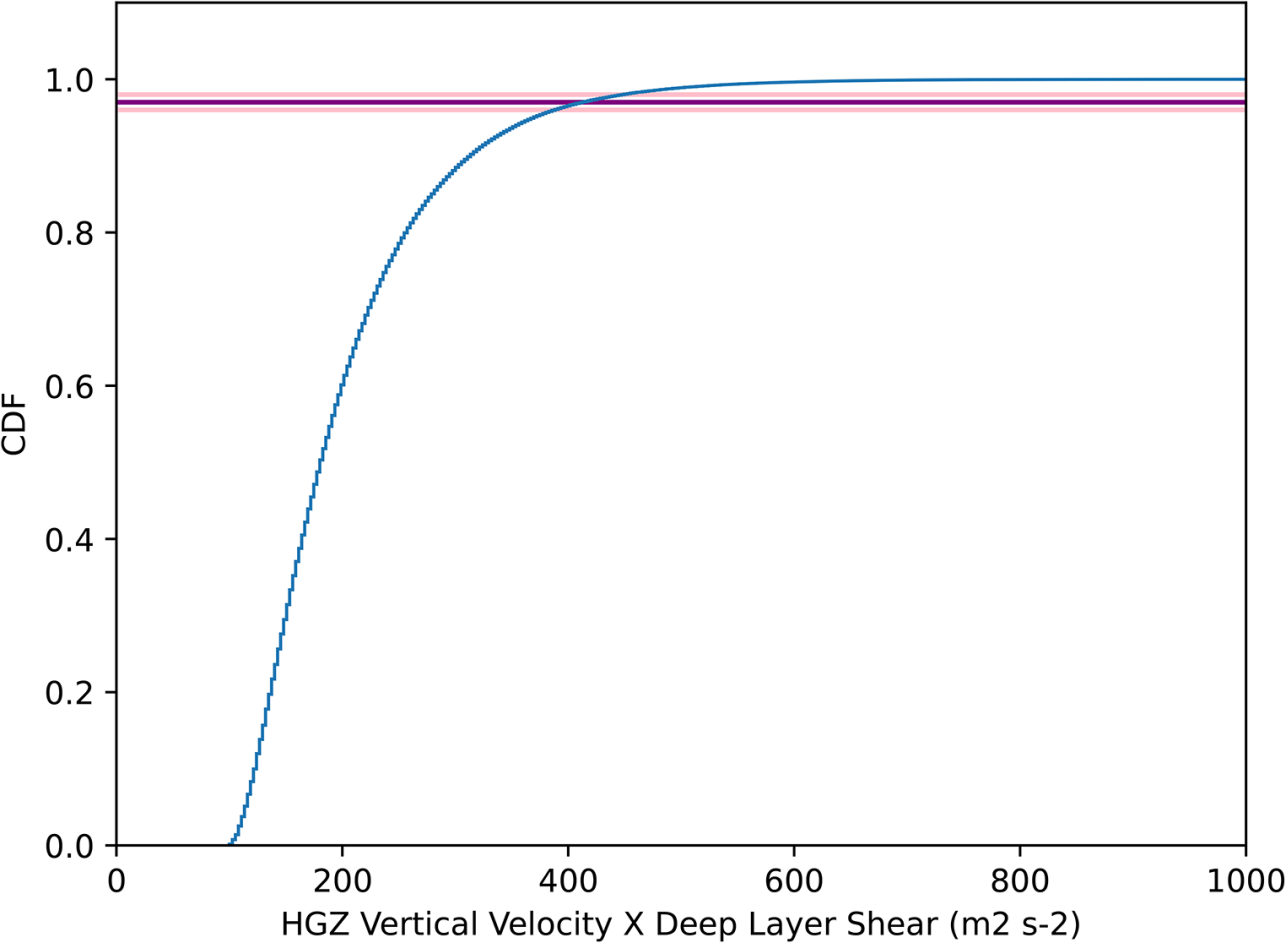
CDF of vertical velocity in the hail growth zone multiplied by deep layer shear (925 − 500 hPa bulk shear) for all 20 years of 2.2 km model data. The 96nd to 98th percentiles are shown as pink lines (97th as purple)

Based on observational studies, 3 to 8% of severe hail (those ≥ 2 cm diameter) is significant severe hail (≥ 5 cm diameter). Recent estimates favour towards the lower end, i.e. 3%, as historical data tend to under-represent marginal cases compared to extreme cases, hence the significant severe-to-severe ratio is skewed towards higher values in earlier studies. In our severe hail potential proxy, the thresholds for the vertical velocity and shear are both 10 m s^− 1^, so that the minimum value for updraft speed × wind shear is 100 m^2^s^− 2^. Higher values could favour significant severe hail production. Considering all instances in the model dataset where the criteria of the severe hail proxy are satisfied, a 3% level corresponds to ∼ 400 m^2^ s^− 2^ (Fig. 4). This can therefore be used as a threshold for significant severe hail for further studies. We understand that the rarity of such hailstorms would mean that most cases would not be captured, and the skill of the proxy for significant severe hail will thus be lower than for severe hail.

### Observational data

The European Severe Storms Laboratory (ESSL) maintains the most extensive dataset of severe storm hazards covering Europe, the European Severe Weather Database (ESWD, Dotzek et al. 2009). The severe hail reports in ESWD are submitted by ESSL staff monitoring local news across Europe, volunteers (including professionals), and by collection from national databases. We use the ESWD severe hail data for Europe for the analysis period March 1999 to February 2019, extracting those with quality assurance code QC0+ (plausibility checked) or higher confidence level and 2 cm maximum hail diameter or greater. The features of the observational dataset will be discussed in the following section, where we compare them with our findings from the model-derived climatology.

## Results

### The CPM-based climatology

**Fig. 5:**
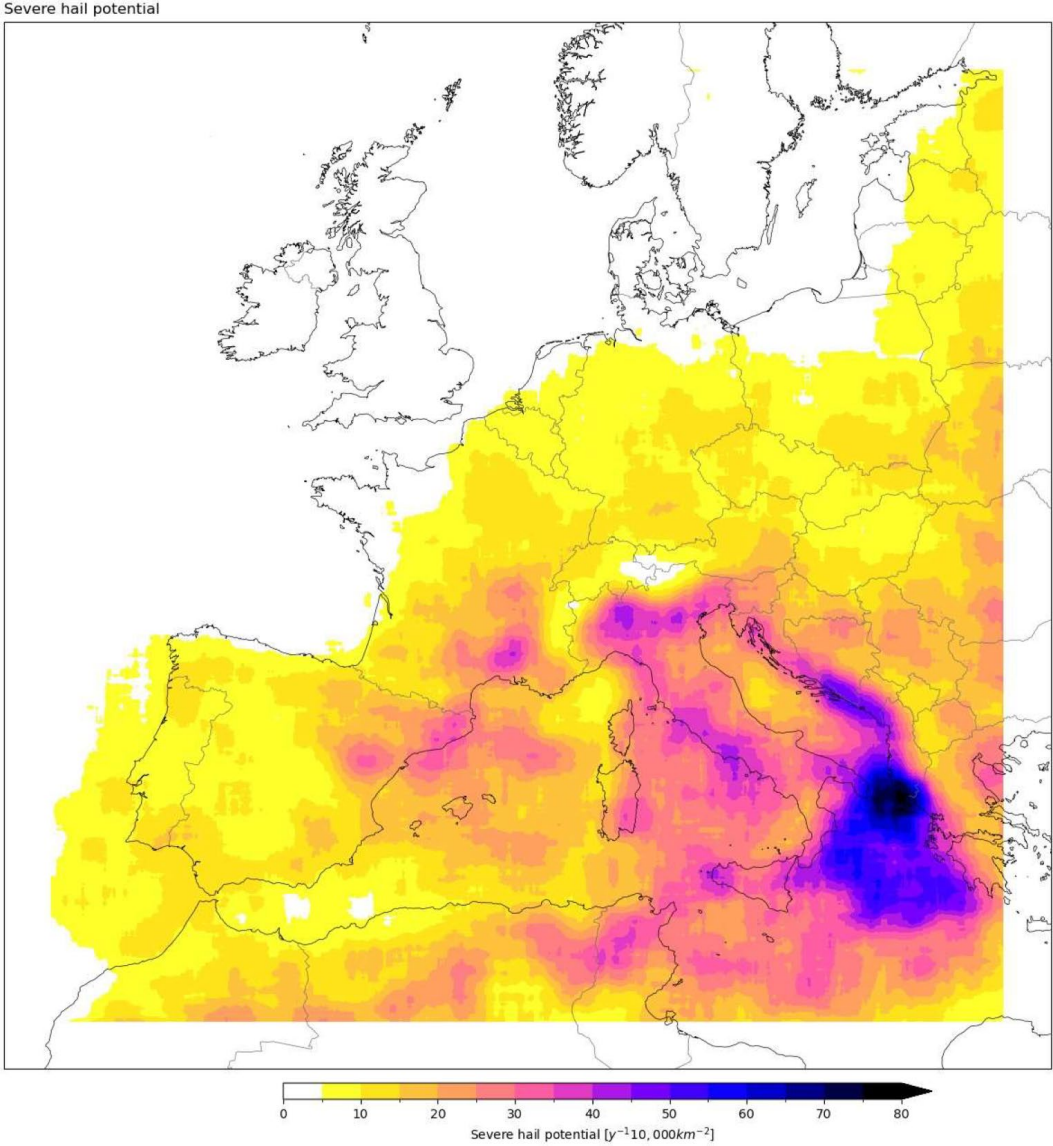
Frequency of severe hail potential in Europe in the 2.2 km model, for 1999–2019. A spatial smoothing is applied by averaging the neighbouring ± 25 grid points in each direction. The map shows the whole domain, with 70 grid points from each direction excluded from the analysis (masked boundaries as white)

The severe hail potential is computed at each grid point in the 20-year simulation data, every 3 h. Then the total at each grid point is divided by 20, to find the annual average frequencies. As these are rare events, we visualise these frequencies per 10,000 km^2^ (i.e. per 100 km × 100 km), by applying a spatial smoothing (Fig. 5). We find frequencies in the range of 0 to 10 y^− 1^ 10,000 km^− 2^ over most land areas, but the frequency exceeds 40 y^− 1^ 10,000 km^− 2^ in some localities, such as parts of Northern Italy (south of the Alps).

Generally, the likelihood of severe hail decreases towards the north of Europe. Hotspots exist in the south, such as Italy, NE Spain, S France, and locally in the Balkans. The British Isles, as well as Scandinavia, have the least severe hail potential. Coastal zones in the north also have very low exposure, such as the Baltic Sea, Brittany and the Channel coasts of France, and the land around the North Sea. The Mediterranean Sea, however, has much higher frequencies of severe hail potential, both along the coasts and offshore. Despite high values in the surrounding region, the high mountains of the Alps themselves have little exposure.

**Fig. 6:**
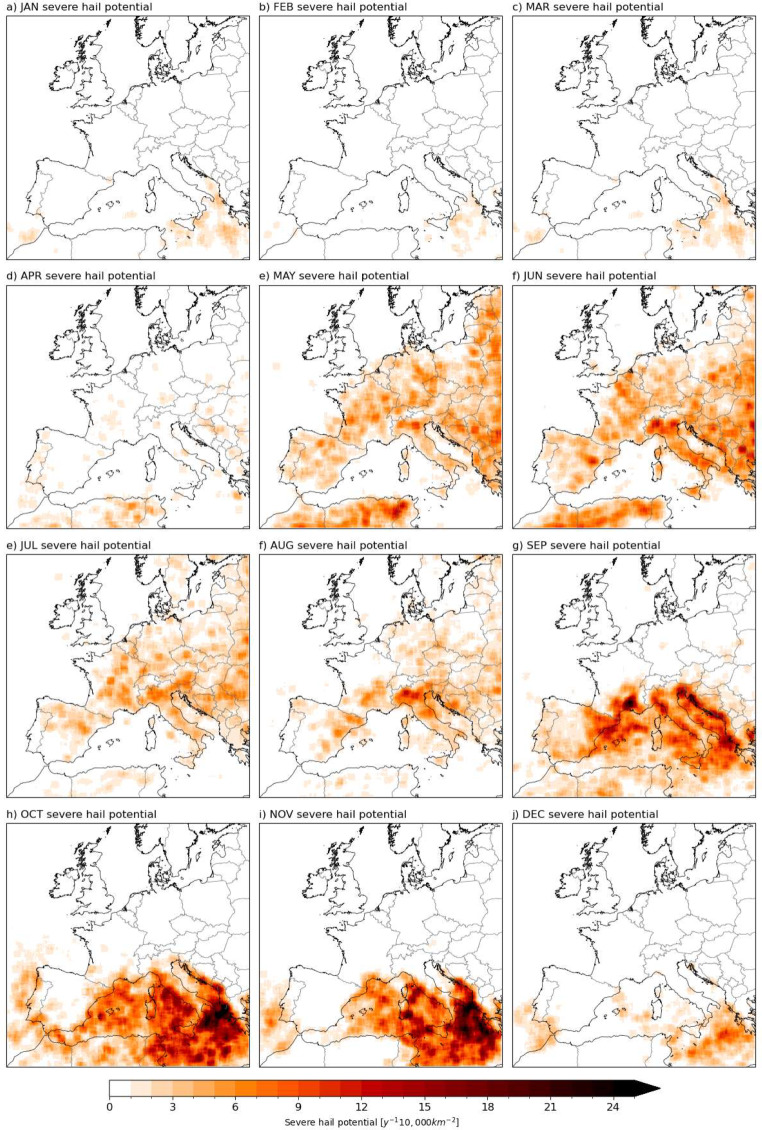
Monthly frequency of severe hail potential across Europe. A spatial smoothing is applied by averaging neighbouring ± 25 grid points in each direction

Looking at the seasonal variation, severe hail potential in Europe is highest from May to August, over most land areas (Fig. 6). The Mediterranean Sea has a higher peak in the autumn, with some local coastal areas having higher frequencies in September and October, and severe hail cases occur in central parts of the Mediterranean Sea during the whole of winter.

**Fig. 7:**
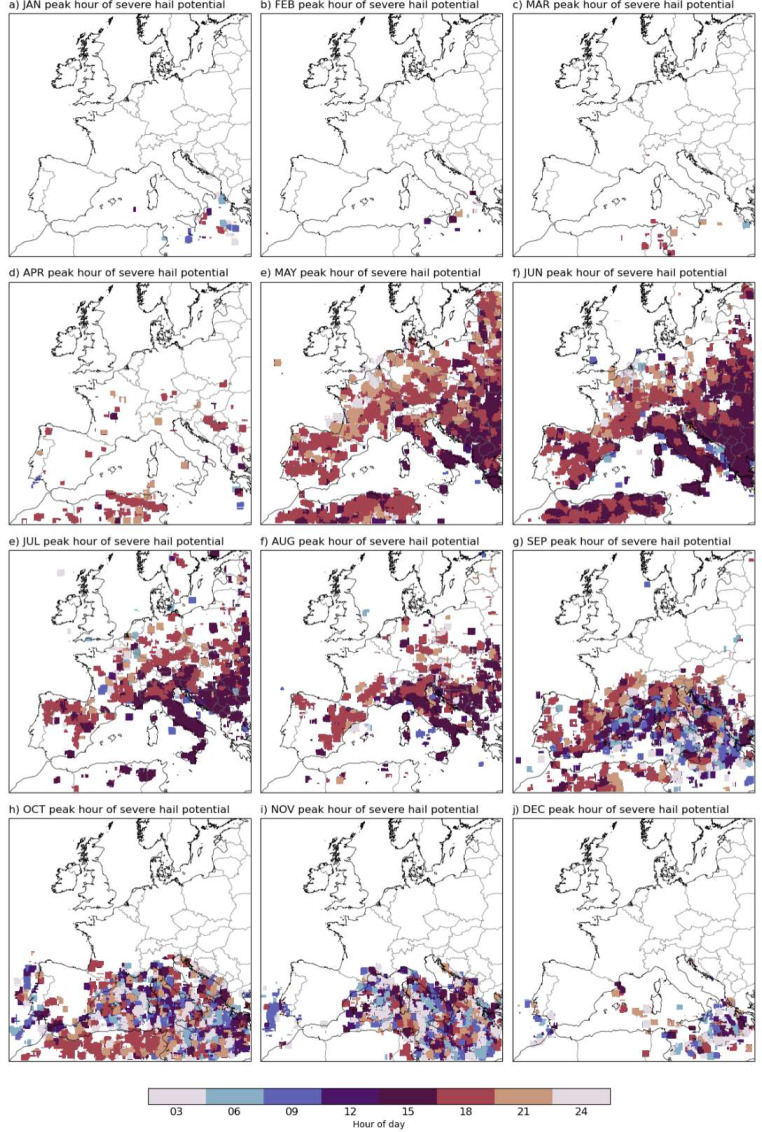
Peak hour of severe hail potential for each month (defined as the most frequent occasion in model time in UTC)

We find that hailstorms over the land follow a diurnal cycle (Fig. 7); they mostly occur from the afternoon into the evening. The slightly earlier hour on initiation in the east of the domain reflects the local solar cycle. By contrast, the Mediterranean Sea and coastal areas can experience severe hail at any time of the day in the autumn and winter, as solar radiation forcing is not the dominant factor, and more non-surface-based convection (elevated thunderstorms) exist. These features are in line with previous severe hail climatologies for Europe, with convection following the solar cycle over land, while this is not the case for sea areas (Púčik et al. 2019; Dessens 1986; Hulton and Schultz 2023; Punge and Kunz 2016; Burcea et al. 2016; Kahraman et al. 2016).

**Fig. 8:**
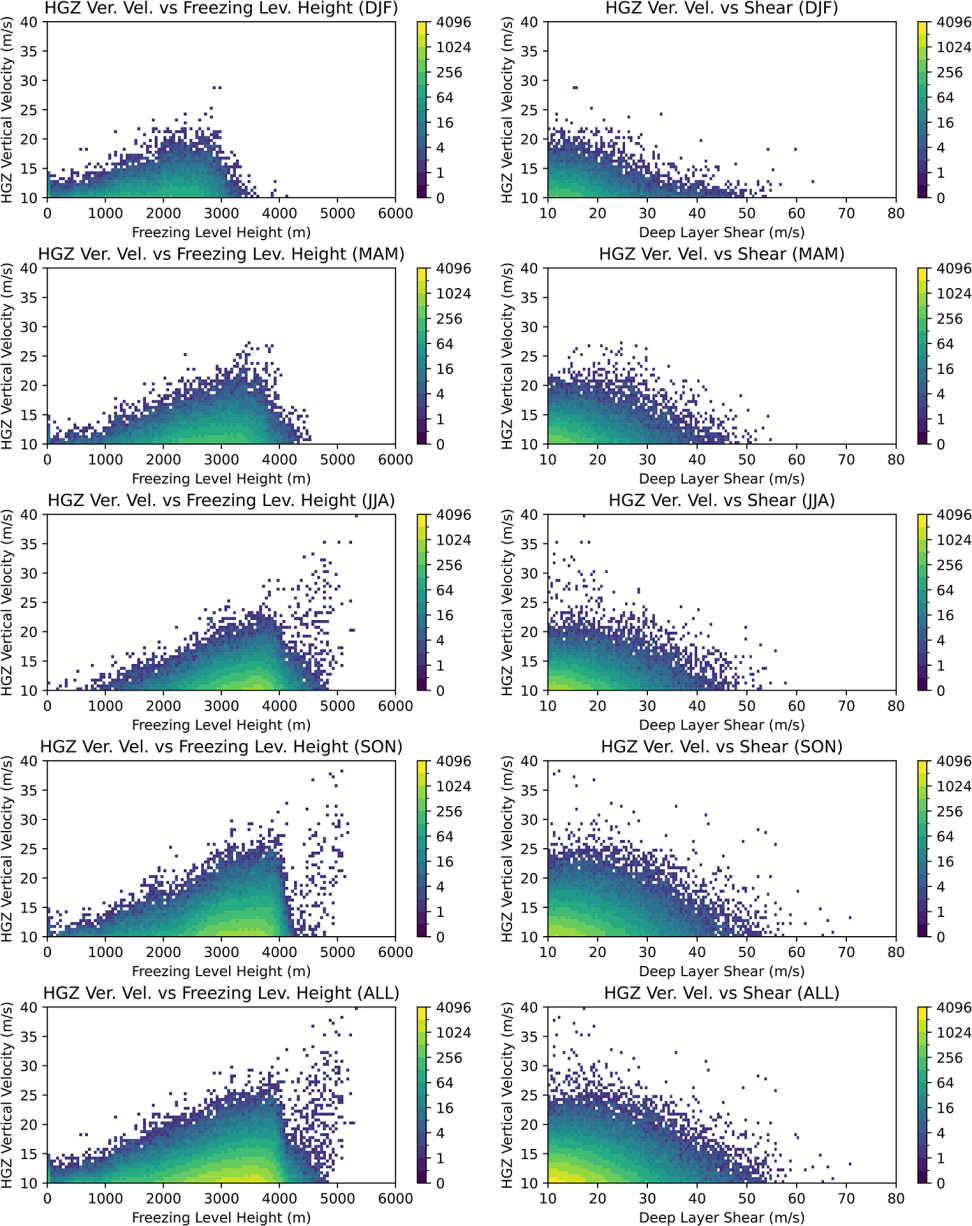
Frequency of Hail Growth Zone vertical velocities (when graupel exists) within freezing level height and deep layer shear bins for four seasons (DJF, MAM, JJA, SON) and the whole year (ALL)

In addition to the spatial, seasonal, and diurnal distributions, it is also important to see how the hail proxy parameters are distributed. We find that stronger updrafts in the HGZ typically occur within warmer storms (Fig. 8). In winter, updrafts are less strong, but the wind shear is stronger. In summer, stronger updrafts are present, but the shear is less strong. These are all expected outcomes for observed European hailstorms, and the distributions indicate realistic features compared to observations (e.g. Taszarek et al. 2020b).

### Comparison with observations and existing climatologies

#### ESWD severe hail reports and severe hail potential

**Fig. 9:**
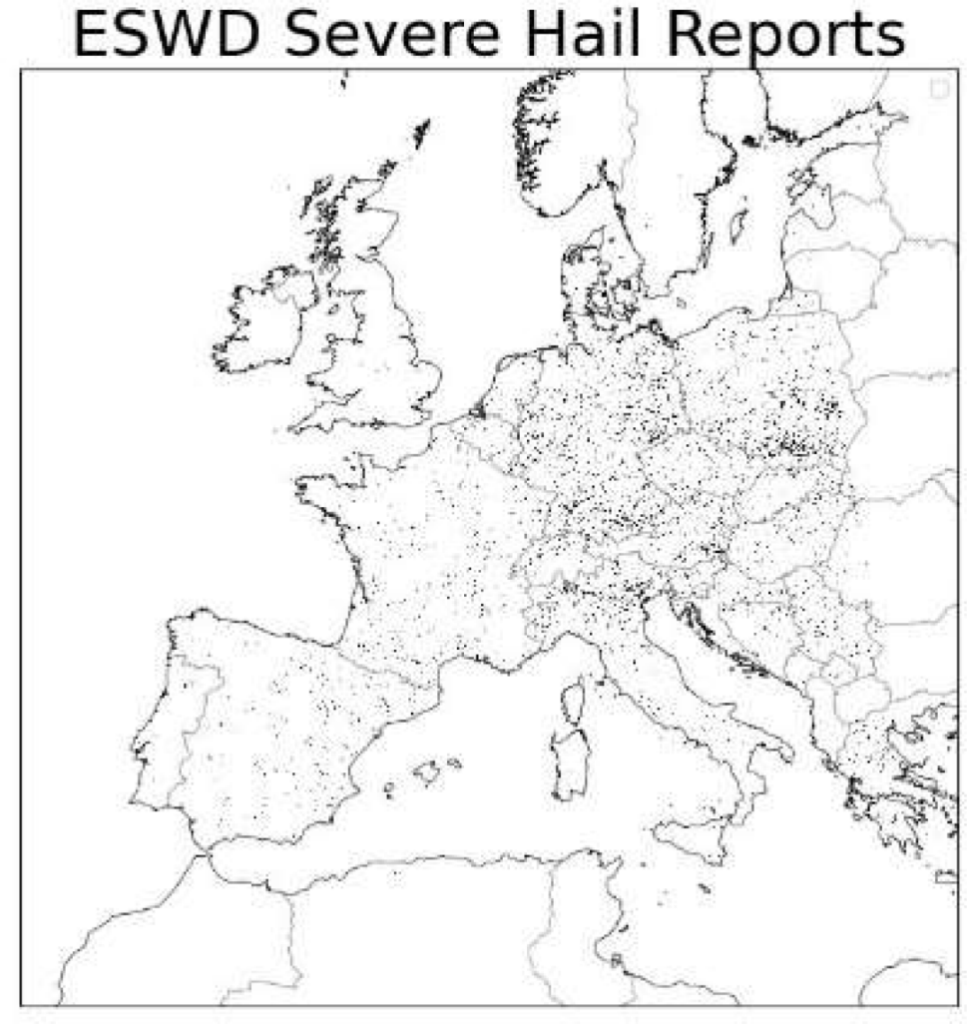
Severe hail reports from ESWD used in the study

We aim to validate our climatology using observations of European severe hail, starting with ESWD observations (Sect. 2.3). It is important to emphasise that severe weather observations are not homogeneous in space and time and should be treated accordingly (Fig. 9). Due to the locality and short time-frame of hailstorms, and varying reporting practices spatially and temporally, a lack of reports does not mean that there are no severe hailstorms. In fact, reporting efficiency (RE) is the main limiting factor for usage of such datasets. Theoretically, the number of reports(for unique events) for a given region over a particular timeframe, R, is

$$\text{R}=\text{R}\text{E}\times \text{E}$$

where E is the number of events that actually occurred, and RE is a number between 0 and 1: the reporting efficiency. Due to various reasons, such as widespread adoption of relevant technology in the recent decade (internet, smart phones, social media, etc.), purposeful efforts (a rise in scientific interest in severe thunderstorms in Europe, international collaborations, establishment of ESWD, etc.), and changes in population (to a lesser extent), the RE can be considered to have increased nonlinearly over time. This overshadows the natural variability of severe hailstorms throughout the decades, which could be order(s) of magnitude lower than the prior, despite the stochastic nature of hailstorms. From the observations only, it is not possible to know if we have reached a RE plateau. Therefore, it is also impossible to detect any trend in the occurrence of hailstorms from observed reports only.

**Fig. 10:**
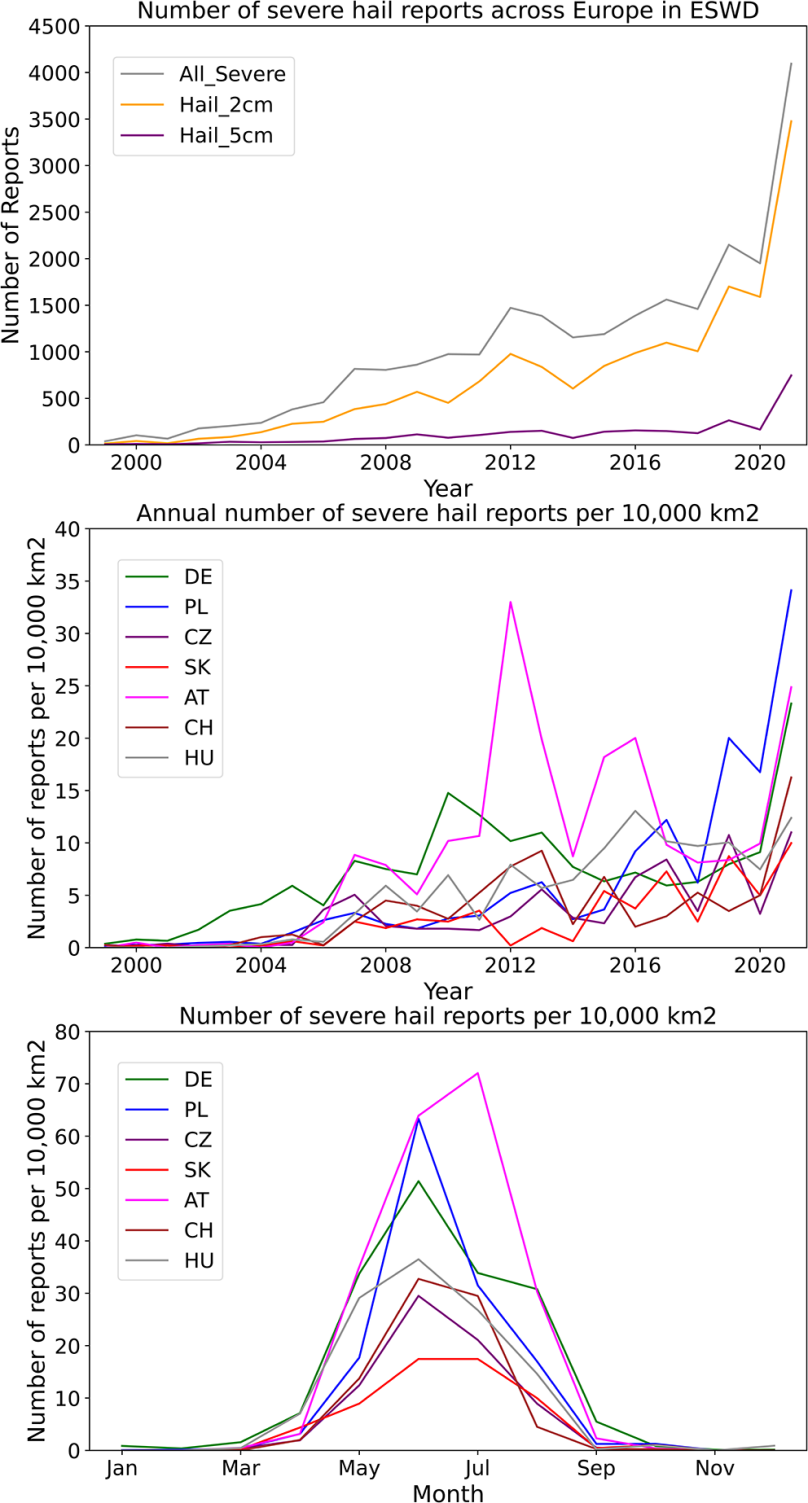
(**a**) Number of ESWD severe hail reports of all hail, ≥ 2 cm, and ≥ 5 cm diameter in Europe from 1999 to 2021. (**b**) Annual number of ESWD severe hail reports per 10,000 km^2^ for 1999–2021 for countries in central Europe. Abbreviations for the countries: DE: Germany, PL: Poland, CZ: Chechia, SK: Slovakia, AT: Austria, CH: Switzerland, HU: Hungary. (**c**) As in b, but for each month

The number of severe hail reports in ESWD increases nonlinearly (Fig. 10a). It is possible the rise in 2021 could be due to an increase in real events but, especially during its early years, the dataset suffers from insufficient RE. It is also important to appreciate different reporting practices in different countries, i.e. the increase of reports in Poland in recent years is likely linked to new volunteers from the Skywarn organization (https://lowcyburz.pl), rather than due to a lack of hailstorms there previously (Fig. 10b).

Despite issues with reporting efficiency, the ESWD dataset may be more reliable for assessing the seasonal variation or diurnal cycle of hail for specific countries. These aspects of the dataset may be useful for the validation of a model-derived climatology. For instance, the annual distribution of severe hail reports in seven selected central European countries indicate a May-to-August peak in severe hailstorms, with almost no occurrence in other months (Fig. 10c). The different geographies will influence the occurrence of hailstorms between the countries, with reporting efficiency (which will be influenced by different population densities) also contributing to differences, e.g., due to larger mountainous areas in Slovakia, Switzerland, etc. Overall, the captured annual distribution by the model + proxy (Fig. 5) agrees well with the observed distribution.

**Fig. 11:**
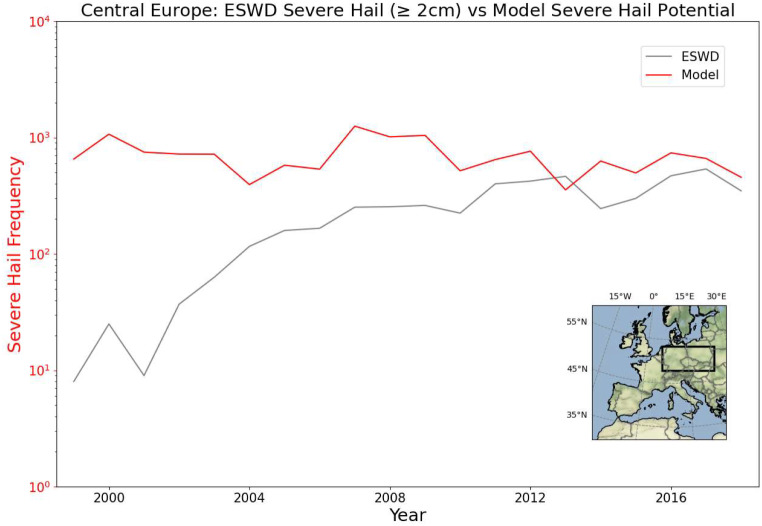
Number of ESWD severe hail reports in Central Europe (≥ 2 cm in diameter) and the frequency of severe hail potential extracted from the model simulation for the same area from 1999 to 2018. Note the logarithmic scale of the y-axis. The analysis area is depicted in the lower right panel by the black box

The model-derived severe hail potential frequency in Central Europe and the number of ESWD reports with severe hail (diameter ≥ 2 cm) converges with time and seem to be comparable from 2010 onwards (Fig. 11). Note that ESWD reports (for the whole of Europe) during this time period have been found to be relatively stable, based on the diurnal cycle of large hail and the time accuracy of reports (Hulton and Schultz 2023). Before 2004 it is likely the dataset is significantly affected by under-reporting issues.

**Fig. 12:**
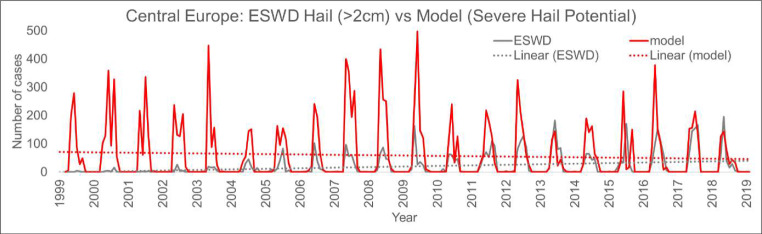
Monthly total number of ESWD severe hail reports and model-derived severe hail potential in Central Europe. A linear trend is shown for both as dashed lines

For a rare and localised weather event like severe hail, it is not meaningful to perform a trend analysis with just two decades of data. But it might be a useful tool to assess whether the observed changes in occurrences are real or due to RE effects. The annual variability of model-derived severe hail potential frequency suggests there is no strong trend within the 20 year simulation period: the overall decrease in 20 years is 27% (Figs. 11 and 12). This could mean that most of the increase in severe hail occurrences in the ESWD dataset stems from changes in reporting practices, rather than from actual increases in occurrences.

Regarding the seasonality of severe hail in Central Europe, the model + proxy seems to capture the May-to-August peaks suggested by the observations (Fig. 12). Note how the observed peaks are of comparable size since around 2010, likely a reflection of improved reporting practices. It is important to note here that a 20-year-long hindcast simulation is expected to capture the main characteristics of the climate. The model has information about the observed state at the boundaries of the European domain, where it is forced by ERA Interim and observed sea surface temperatures. However, in the interior of the model domain, the model evolves freely and thus the individual occurrence of hailstorms on a month-by-month basis is not expected to absolutely match the observations, although good agreement may be seen in situations where hailstorm occurrence is strongly constrained by the large-scale conditions.

**Fig. 13:**
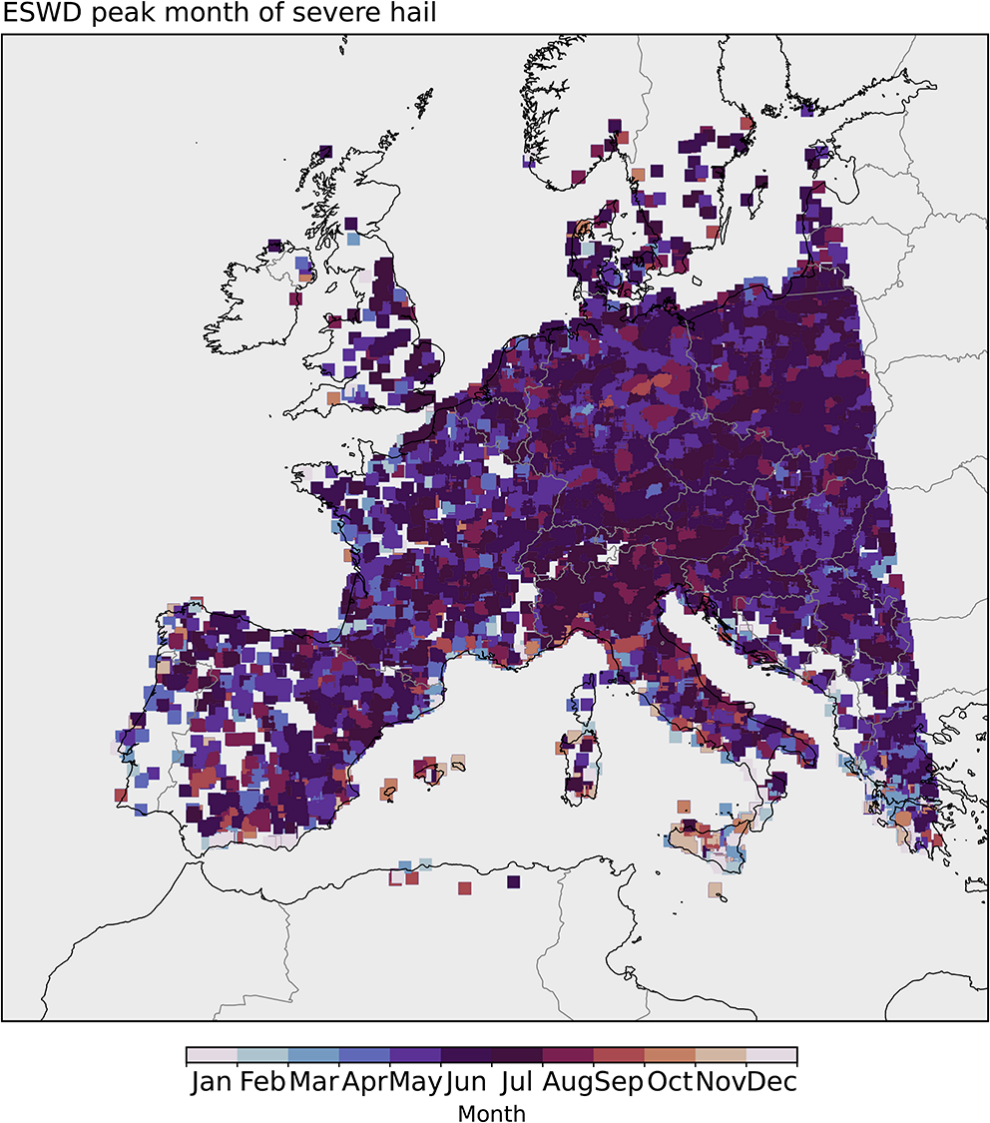
Peak month of severe hail based on the European Severe Weather Database reports. Data is for ≥ 2 cm diameter hail, between 1999 March and 2019 February. A mask is applied where there are fewer than 0.001 reports per km^2^. Note that a spatial smoothing is applied by averaging neighbouring ± 10 grid points in each direction

**Fig. 14:**
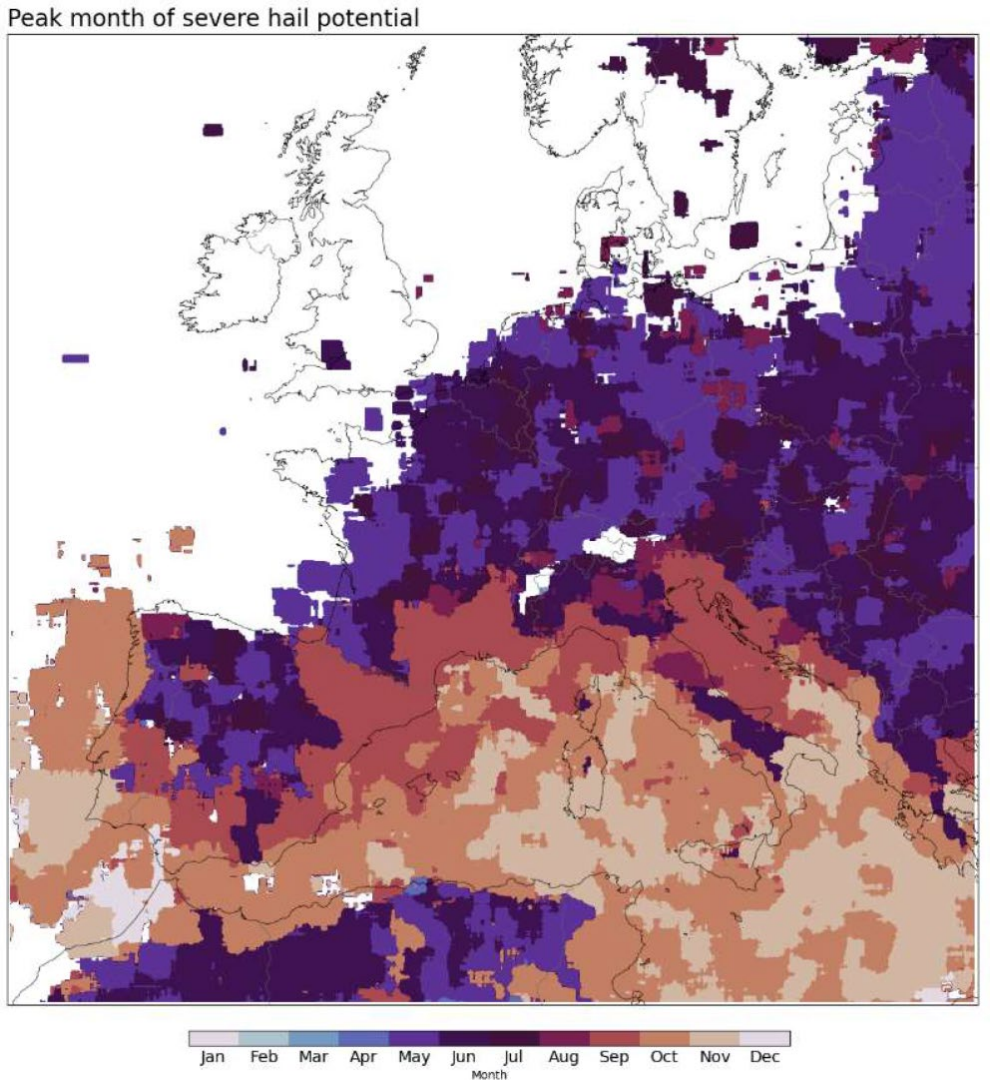
Peak month of severe hail potential based on the model data. The underlying hindcast data is from March 1999 to February 2019. A mask is applied where there are fewer than 0.00015 cases per km^2^. Note that a spatial smoothing is applied by averaging neighbouring ± 20 grid points in each direction

Compared with our model results, severe hail observations from ESWD also indicate a peak severe hail month of May to August for most of the European continent, while the Mediterranean islands and some coastal zones have autumn or even, locally, winter peaks (Fig. 13). We note that there are a very limited number of reports offshore. For comparison, a similar map is produced for peak severe hail potential month based on the model data (Fig. 14). Generally, continental Europe shows similar peak months to the ESWD observations, but autumn peaks (mostly September) exist around the Mediterranean coasts. Some of this comes from spatial smoothing, but the algorithm extracts more autumn Mediterranean storms with severe hail potential compared to those over land in the summer. It is hard to assess if this is the case in reality, with only reporting-based observational data. However, satellite-based studies indicate a severe hail peak over the Central Mediterranean in the autumn (Bang and Cecil 2019; Bedka et al. 2023). Hail occurs every 3 to 5 days on average, at meteorological stations in the Ionian Islands of Greece, and almost all hail days on the western coasts of Greece happen between October-March rather than during the warm season (Sioutas 2019).

#### Comparison with other climatologies

In Europe, hail has conventionally been studied nationally or locally, and with many different approaches such as different size thresholds, or event descriptions. This makes it hard to compare the outputs from different studies, so here we focus more on larger-scale studies, but we also include comparison to national climatologies.

Warm season radar-derived hail frequencies for France, Germany, Belgium, and Luxembourg between 2005 and 2014 indicate the peak areas for hail days as the Massif Central and eastern France, as well as southern Germany, among the regions studied (Fluck et al. 2021). These areas are also hotspots in our model + proxy (Fig. 5). Furthermore, the model + proxy is able to capture the location of the region with the highest average number of hail days in the warm season, which is on the leeward side of the Massif Central summits (Fig. 6; the Fluck et al. 2021 study links this to low-level convergence in addition to the location of mountains). However, the high number of cases slightly south of this region in our model in September does not exist in the radar-derived hail frequencies. The radar-derived diurnal distribution is at its maximum between 14:00 and 18:00 local time, which is consistent with peak frequencies at around 18z in our results. Small spatial features such as the later hour of hailstorms in SW France are also captured by our model + proxy (Fig. 7e and f). The overall spatial distribution in France also aligns well with a hail risk map produced using hail measurement networks and insurance-based hail damage data (Vinet 2001), which shows high risk around the center-east, Southern Alps and southwest, and gradually decreasing risk towards the northwest.

A proxy developed for estimating damaging hail hazard using 6-hourly ERA-Interim (0.75°) reanalysis data between 1979 and 2015 suggested June, July, and August as the peak months for large hail frequency over most of the continent, and September-October for coastal areas in the south and west (Prein and Holland 2018), agreeing well with our results. The spatial distribution is also essentially similar, revealing higher intensities from E Spain through S and E France, Italy, and locally in the Balkans, although this previous study lacks the fine-scale detail provided by our study (Prein and Holland 2018). An interesting aspect of the Prein and Holland (2018) results are the higher peaks over parts of the Mediterranean Sea (C and W Mediterranean and Adriatic) compared to over European land areas, confirming our spatial patterns over the sea (Fig. 5). A follow-up ERA5-based study (Torralba et al. 2023) focused on Italy with similar outcomes, but providing further details such as south of the Alps in northern Italy having a severe hail maximum (up to 3–5 hail days in June), just as our proxy highlights. They also capture up to 3–4 hail days in October along a narrow coastline in SW Italy, which is in line with our findings, and a preliminary indicator for what is happening offshore in autumn. An earlier observational study on Italian hailstorms showed similar distributions as well, but higher frequency in the northwest, although the NCDC data shows the northeast as the hotspot (Baldi et al. 2014).

An observational study by Santos and Belo-Pereira (2019) suggest that Portugal has roughly a north-to south gradient in hailstorm frequency, with Porto having the highest frequency (2.6 hail days per year). Overall, there is a spring peak and an autumn peak of convection in the country, with April being the peak hail month. Our results show a spring peak in the north of the country, but not near to the coastline (Fig. 14), Either the winter storms in northern Portugal produce smaller hail that our proxy is not tuned for (which is possible as some of these storms occur without thunder, based on Santos and Belo-Pereira 2019), or there is a negative bias in our results for winter, and an overestimation for the autumn. For the south of the country, however, the hail frequency is lower (down to less than 0.5 days per year), and the seasonality of thunderstorms indicate autumn to be more favourable (Santos and Belo-Pereira 2019). Hail-producing supercells in Spain occur between June-October, mostly around the east and northeast (Calvo-Sancho et al. 2022), which our results confirm for severe hail potential, with a slight leaning towards the autumn, especially near to the coasts, although their results have the bulk of the storms in August.

Hailstorms in Croatia have a diverse seasonal distribution, with the immediate coastline having most of the occurrences in winter, inner parts in warm season, and the transient region just slightly away from the coast for almost the whole year (Blašković et al. 2023). The highest frequency is 4.8 days per year, on a mountain which is not far away from the coast in the west, and recognized as a hotspot in our hail frequency map (Fig. 5). The second highest frequency is observed further south near to the coast, as 3.8 days per year (Blašković et al. 2023), where our proxy has comparable values, becoming higher further south. The high-resolution topography in the model could have helped with capturing these details. Further east, to the Balkans, Serbia has the highest frequency in the western mountains, with 3.4 days per year (Ćurić and Janc 2016). They mostly occur from April to August, as our proxy also finds.

The British Isles are known to experience hailstorms less often than further south in Europe. The highest number of hailstorms exceeding 15 mm diameter is observed in central and eastern England, with a frequency of “over 15” per 1000 km^2^ per 100 years (Webb et al. 2009), which is well below most of continental Europe. Our severe hail potential shows similar values (all lower than 50).

Punge et al. (2017) combined satellite data (overshooting tops of thunderstorms) and ERA-Interim to produce a hail frequency map for Europe, which also highlighted similar areas of severe hail in N Italy, SE Austria, E Spain and the Massif Central. They also found a peak in NE Algeria and N Tunisia, an area also highlighted by our model, albeit near our domain border.

A 4-year satellite-based study has highlighted that the Central Mediterranean experiences up to 4–6 hail occurrences per year, which is higher than anywhere over the European landmass (Bang and Cecil 2019). This is in line with our model + proxy predictions of autumn storms offshore, which cannot be validated by ground-based observations. Although satellite-based estimates might not reflect the ground truth completely, it is encouraging to retrieve similar results via convection-permitting model output and our derived proxy. This marine region might be a little-known alley for hailstorms in Europe.

Overall, our proxy is largely giving values in good agreement with other climatologies, although with potential biases in some regions such as parts of Mediterranean coasts.

## Conclusion

Our CPM-based severe hail climatology for Europe is the first benefiting from simulated convection, and provides results consistent with current knowledge of the distribution of European hailstorms, and adds value as follows:

1. The model and severe hail potential proxy is an objective measure and does not depend on observational biases such as variable reporting efficiencies through space and time. Hence, less densely populated areas, less vulnerable locations, regions without good satellite/radar coverage, and night-time storms, etc. are equally and fairly represented in the climatology built using the CPM output. It can be used to study any trends if run for longer periods, and provides information on the occurrence of hailstorms over the sea, although models have their own limitations (e.g. location-based biases), which should be considered in the analyses.
2. Although a 2.2 km horizontal grid spacing is not sufficient to resolve the in-storm dynamics of a hailstorm, features such as the updraft speed in the hail growth zone are directly simulated in a CPM, rather than indirectly estimated via metrics like CAPE, as in coarse resolution models. This enables the development of a better “ingredients-based” proxy than others built on large-scale hailstorm-favouring environments only. Additionally, better representation of the topographical details, improved microphysics, and simulation of finer scale features of atmospheric flow by CPMs are crucial in generating thunderstorms. Thus, our CPM-based severe hail measure provides a more reliable estimate of hailstorm occurrence than previous proxies based on coarser-resolution traditional climate models.
3. The CPM can be run not only for the current climate, but also for future projections. This will allow us to perform a similar analysis for a changing climate, i.e. to examine future changes in severe hailstorms: currently one of the most uncertain aspects of global warming.

Overall, the climatology built by a CPM-dedicated proxy performs well, and represents a significant advance compared to previous studies. However, we acknowledge that going further is possible. The use of thunderstorm-tracking tools (based on more frequent precipitation/reflectivity output), the analysis of updraft width via multiple neighbouring grids, the use of parameters focused on thunderstorm phase and morphology, better representing hail in microphysics schemes, using a coupled hail model, updraft helicity, etc., are all potential tools for performing an even more advanced analysis, and are subject to future studies.

## Data Availability

European 2.2 km model dataset can be used under licence from the Met Office, but restrictions to the use apply, and must respect the work plans of EUCP project partners and of CORDEX-FPS-Convection. ESWD data is available from ESSL (www.eswd.eu).

## References

[CR1] Adams-Selin RD, Ziegler CL (2016) Forecasting hail using a one-dimensional hail growth model within WRF. Monthly WeatherReview 144(12):4919–4939. 10.1175/MWR-D-16-0027.1

[CR3] Allen JT, Tippett MK (2015) : The characteristics of United States hail reports: 1955–2014. Electron J Severe Storms Meteor, 10 (3), https://ejssm.com/ojs/index.php/site/article/view/60

[CR2] Allen JT, Giammanco IM, Kumjian MR, Punge HJ, Zhang Q, Groenemeijer P et al (2020) Understanding hail in the Earth system. Rev Geophys 58(e2019RG000665). 10.1029/2019RG000665

[CR4] Baldi M, Ciardini V, Dalu JD, Filippis TD, Maracchi G, Dalu G (2014) Hail occurrence in Italy: towards a national database and climatology. Atmos Res 138:268–277

[CR5] Ban N, Caillaud C, Coppola E et al (2021) The first multi-model ensemble of regional climate simulations at kilometer-scale resolution, part I: evaluation of precipitation. Clim Dyn 57:275–302. 10.1007/s00382-021-05708-w

[CR6] Bang SD, Cecil DJ (2019) Constructing a Multifrequency Passive Microwave Hail Retrieval and Climatology in the GPM Domain. J Appl Meteor Climatol 58:1889–1904. 10.1175/JAMC-D-19-0042.1

[CR7] Bedka KM, Allen JT, Punge HJ, Kunz M, Simanovic D (2018) A long-term overshooting convective cloud-top detection database over Australia derived from MTSAT Japanese advanced meteorological imager observations. J Appl Meteor Climatol 57:937–951. 10.1175/JAMC-D-17-0056.1

[CR8] Bedka K, Scarino B, Itterly K, Spangenberg D, Homeyer C, Allen J, Bang S, Cecil D (2023) Toward the development of Hailstorm climatologies Derived from reanalyses and Infared/Passive Microwave Satellite Imagers. 11th Eur Conf Severe Storms. 10.5194/ecss2023-174. ECSS2023-174, Bucharest, Romania, 8–12 May 2023

[CR9] Berthou S, Kendon EJ, Chan SC et al (2020) Pan-european climate at convection-permitting scale: a model intercomparison study. Clim Dyn 55:35–59. 10.1007/s00382-018-4114-6 10.1007/s00382-020-05192-8 PMC7370986 32713994

[CR10] Best MJ, Pryor M, Clark DB, Rooney GG, Essery RLH, Ménard CB, Edwards JM, Hendry MA, Porson A, Gedney N, Mercado LM, Sitch S, Blyth E, Boucher O, Cox PM, Grimmond CSB, Harding RJ (2011) The joint UK land environment simulator (JULES), model description—part 1: energy and water fluxes. Geosci Model Dev 4:677–699. 10.5194/gmd-4-677-2011

[CR11] Billet J, DeLisi M, Smith BG (1997) Use of regression techniques to predict hail size and the probability of large hail. Wea Forecast 12:154–164

[CR12] Blašković L, Jelić D, Malečić B, Omazić B, Güttler I, Telišman Prtenjak M (2023) Trend analysis and climatology of hail in Croatia. Atmos Res 294:106927. 10.1016/j.atmosres.2023.106927

[CR16] Boutle IA, Eyre JEJ, Lock AP (2014) Seamless stratocumulus simulation across the turbulent gray zone. Mon Weather Rev 142:1655–1668. 10.1175/MWR-D-13-00229.1

[CR13] Brimelow JC, Reuter GW, Poolman ER (2002) Modeling Maximum Hail size in Alberta thunderstorms. Wea Forecast 17:1048–1062. 10.1175/1520-0434(2002)017%3C1048:MMHSIA%3E2.0.CO;2

[CR14] Brimelow JC, Burrows WR, Hanesiak JM (2017) The changing hail threat over North America in response to anthropogenic climate change. Nat Clim Change 7(7):516–522

[CR85] Browning KA (1964) Airflow and precipitation trajectories within severe local storms which travel to the right of the winds. J Atmos Sci 21:634–639. 10.1175/1520-0469(1964)0212.0.CO;2

[CR15] Browning K, Foote G (1976) Air flow and hail growth in supercell storms and some implications for hail suppression. Q J R Meteorol Soc 102(433):499–533. 10.1002/qj.49710243303

[CR17] Burcea S, Cică R, Bojariu R (2016) Hail climatology and trends in Romania: 1961–2014. Mon Wea Rev 144:4289–4299. 10.1175/MWR-D-16-0126.1

[CR18] Calvo-Sancho C, Díaz-Fernández J, Martín Y, Bolgiani P, Sastre M, González-Alemán JJ, Santos-Muñoz D, Farrán JI, Martín ML (2022) Supercell convective environments in Spain based on ERA5: hail and non-hail differences. Weather Clim Dynam 3:1021–1036. 10.5194/wcd-3-1021-2022

[CR19] Cerveny RS et al (2017) WMO assessment of weather and climate mortality extremes: Lightning, tropical cyclones, tornadoes, and hail. Wea Clim Soc 9:487–497. 10.1175/WCAS-D-16-0120.1

[CR20] Chapell CF, Rodgers DM (1988) Meteorological Analysis of the Cheyenne, Wyoming, Flash Flood and Hailstorm of 1 August 1985. NOAA Technical Report ERL 435-FSL 1. U.S. Department of Commerce NOAA ERL. https://repository.library.noaa.gov/view/noaa/11264/noaa_11264_DS1.pdf

[CR21] Clark DB, Mercado LM, Sitch S, Jones CD, Gedney N, Best MJ, Pryor M, Rooney GG, Essery RLH, Blyth E, Boucher O, Harding RJ, Huntingford C, Cox PM (2011) The joint Uk land environment simulator (jules), model description—part 2: carbon fluxes and vegetation dynamics. Geosci Model Dev 4:701–722. 10.5194/gmd-4-701-2011

[CR22] Coppola E, Sobolowski S, Pichelli E et al (2020) A first-of-its-kind multi-model convection permitting ensemble for investigating convective phenomena over Europe and the Mediterranean. Clim Dyn 55:3–34. 10.1007/s00382-018-4521-8

[CR23] Ćurić M, Janc D (2016) Hail climatology in Serbia. Int J Climatol 36:3270–3279. 10.1002/joc.4554

[CR24] Dee DP, Uppala SM, Simmons AJ, Berrisford P, Poli P, Kobayashi S, Andrae U, Balmaseda MA, Balsamo G, Bauer P, Bechtold P, Beljaars ACM, van de Berg L, Bidlot J, Bormann N, Delsol C, Dragani R, Fuentes M, Geer AJ, Haimberger L, Healy SB, Hersbach H, Hölm EV, Isaksen L, Kallberg P, Köhler M, Matricardi M, McNally AP, Monge-Sanz BM, Morcrette JJ, Park PK, Peubey C, de Rosnay P, Tavolato C, Thépaut JN, Vitart F (2011) The ERA-Interim reanalysis: configuration and performance of the data assimilation system. Q J R Meteorol Soc 137(656):553–597. 10.1002/qj.828

[CR25] Dennis EJ, Kumjian MR (2017) The impact of vertical wind shear on hail growth in simulated supercells. J Atmos Sci 74:641–663. 10.1175/JAS-D-16-0066.1

[CR26] Dessens J (1986) Hail in southwestern France. Part I: hailfall characteristics and hailstrom environment. J Clim Appl Meteor 25:35–47. 10.1175/1520-0450(1986)025%3C0035:HISFIH%3E2.0.CO;2

[CR27] Doswell C (1982) Thermodynamic analysis procedures at the national severe storms forecast center, in Conference on Weather Forecastingand Analysis, 9 th, Seattle, WA (pp. 304–309). Boston, MA: American Meteorological Society

[CR28] Dotzek N, Groenemeijer P, Feuerstein B, Holzer AM (2009) Overview of ESSL’s severe convective storms research using the European severe Weather Database ESWD. Atmos Res 93:575–586

[CR29] Edwards JM, Slingo A (1996) Studies with a flexible new radiation code. I: choosing a configuration for a large-scale model. Q J R Meteorol Soc 122:689–720

[CR30] Edwards R, Thompson RL (1998) Wea Forecast 13:277–285. 10.1175/1520-0434(1998)013%3C0277:NCOHSW%3E2.0.CO;2. Nationwide Comparisons of Hail Size with WSR-88D Vertically Integrated Liquid Water and Derived Thermodynamic Sounding Data

[CR31] Fluck E, Kunz M, Geissbuehler P, Ritz SP (2021) Radar-based assessment of hail frequency in Europe. Nat Hazards Earth Syst Sci 21:683–701. 10.5194/nhess-21-683-2021

[CR87] Foote GB (1984) A study of hail growth utilizing observed storm conditions. J Appl Meteor Climatol 23:84–101. 10.1175/1520-0450(1984)0232.0.CO;2

[CR32] Fraile R, Sánchez J, de la Madrid J et al (1999) Some results from the Hailpad Network in León (Spain): noteworthy correlations among Hailfall parameters. Theor Appl Climatol 64:105–117. 10.1007/s007040050115

[CR33] Grahame N, Riddaway B, Eadie A, Hall B, McCallum E (2009) Exceptional hailstorm hits Ottery St Mary on 30 October 2008. Weather 64:255–263. 10.1002/wea.458

[CR34] Groenemeijer PH, van Delden A (2007) Sounding-derived parameters associated with large hail and tornadoes in the Netherlands. Atmos Res 83:473–487. 10.1016/j.atmosres.2005.08.006

[CR83] Heymsfield AJ, Musil DJ (1982) Case study of a hailstorm in Colorado. Part II: Particle growth processes at mid-levels deduced from in-situ measurements. J Atmos Sci 39:2847–2866. 10.1175/1520-0469(1982)0392.0.CO;2

[CR84] Heymsfield AJ (1983) A technique for investigating graupel and hail development. J Appl Meteor Climatol 22:1143–1160. 10.1175/1520-0450(1983)0222.0.CO;2

[CR35] Houze RA (2012) Orographic effects on precipitating clouds. Rev Geophys 50:RG1001. 10.1029/2011RG000365

[CR36] Hulton F, Schultz DM (2023) Climatology of large Hail in Europe: characteristics of the European severe Weather Database. EGUsphere [preprint]. 10.5194/egusphere-2023-176

[CR37] Johnson AW, Sugden KE (2014) Evaluation of sounding-derived thermodynamic and wind-related parameters associated with large hail events. Electron J Severe Storms Meteorol, 9(5)

[CR38] Kahraman A, Tilev-Tanriover Ş, Kadioglu M, Schultz DM, Markowski PM (2016) Severe hail climatology of Turkey. Mon Wea Rev 144:337–346. 10.1175/MWR-D-15-0337.1

[CR39] Kahraman A, Kadioglu M, Markowski PM (2017) Severe convective storm environments in Turkey. Mon Wea Rev 145:4711–4725. 10.1175/MWR-D-16-0338.1

[CR41] Kendon E, Roberts N, Fowler H et al (2014) Heavier summer downpours with climate change revealed by weather forecast resolution model. Nat Clim Change 4:570–576. 10.1038/nclimate2258

[CR42] Kendon EJ, Fischer EM, Short CJ (2023) Variability conceals emerging trend in 100 year projections of UK local hourly rainfall extremes. Nat Commun 14:1133. 10.1038/s41467-023-36499-9 36882408 10.1038/s41467-023-36499-9 PMC9992391

[CR43] Kitzmiller DH, Breidenbach JP (1993) : Probabilistic nowcasts of large hail based on volumetric reflectivity and storm environment characteristics. Preprints, 26th Int. Conf. on Radar Meteorology, Norman, OK, Amer. Meteor. Soc., 157–159

[CR45] Knight CA, Knight NC (1970) Hailstone embryos. J Atmos Sci 27:659–666. 10.1175/1520-0469(1970)027%3C0659:HE%3E2.0.CO;2

[CR44] Knight CA, Ehhalt DH, Roper N, Knight NC (1975) Radial and tangential variation of deuterium in hailstones. J Atmos Sci 32(10):1990–2000. 10.1175/1520-0469(1975)032%3C1990:RATVOD%3E2.0.CO;2

[CR46] Kumjian MR (2013) Principles and applications of dual-polarization weather radar. Part I: description of the polarimetric radar variables. J Oper Meteor 1(19):226–242. 10.15191/nwajom.2013.0119

[CR47] Kumjian MR, Lombardo K (2020) A Hail Growth Trajectory Model for exploring the Environmental controls on Hail size: Model Physics and Idealized tests. J Atmos Sci 77:2765–2791. 10.1175/JAS-D-20-0016.1

[CR48] Kumjian MR, Lombardo K, Loeffler S (2021) The evolution of Hail production in simulated supercell storms. J Atmos Sci 78:3417–3440. 10.1175/JAS-D-21-0034.1

[CR49] Kunz M, Puskeiler M (2010) High-resolution assessment of the hail hazard over complex terrain from radar and insurance data. Meteor Z 19:427–439. 10.1127/0941-2948/2010/0452

[CR50] Manzato A (2012) Hail in Northeast Italy: Climatology and Bivariate Analysis with the sounding-derived indices. J Appl Meteor Climatol 51:449–467. 10.1175/JAMC-D-10-05012.1

[CR51] Merino et al (2014) Hailstorms in southwestern France: incidence and atmospheric characterization. Atm Res 140–141:61–75. 10.1016/j.atmosres.2014.01.015

[CR86] Miller LJ, Fankhauser JC (1983) Radar echo structure, air motion and hail formation in a large stationary multicellular thunderstorm. J Atmos Sci 40:2399–2418. 10.1175/1520-0469(1983)0402.0.CO;2

[CR52] Mohr S, Kunz M (2013) Recent trends and variabilities of convective parameters relevant for hail events in Germany and Europe. Atmos Res 123:211–228. 10.1016/j.atmosres.2012.05.016

[CR53] Mohr S, Kunz M, Geyer B (2015) Hail potential in Europe based on a regional climate model hindcast. Geophys Res Lett 42(904–10,912). 10.1002/2015GL067118

[CR54] Morgan GM (1972) On the growth of large hail. Mon Weather Rev 100(3):196–205. 10.1175/1520-0493(1972)100%3C0196:OTGOLH%3E2.3.CO;2

[CR88] Musil DJ, Heymsfield AJ, Smith PL (1986) Microphysical characteristics of a well-developed weak echo region in a high plains supercell thunderstorm. J Appl Meteor Climatol 25:1037–1051. 10.1175/1520-0450(1986)0252.0.CO;2

[CR55] Nisi L, Hering A, Germann U, Martius O (2018) A 15-year hail streak climatology for the Alpine region. Quart J Roy Meteor Soc 144:1429–1449. 10.1002/qj.3286

[CR56] Ortega KL (2018) : Evaluating multi-radar, multi-sensor products for surface hailfall diagnosis. Electron J Severe Storms Meteor, 13 (1), http://www.ejssm.org/ojs/index.php/ejssm/article/viewArticle/163

[CR57] Prein AF et al (2015) A review onregional convection-permitting climatemodeling: demonstrations, prospects,and challenges. Rev Geophys 53:323–361. 10.1002/2014RG000475 27478878 10.1002/2014RG000475 PMC4949718

[CR59] Prein AF, Rasmussen RM, Wang D, Giangrande SE (2021) Sensitivity of organized convective storms to model grid spacing in current and future climates. Philosophical Trans Royal Soc A 379(2195):20190546 10.1098/rsta.2019.0546 PMC7934897 33641457

[CR58] Prein and Holland (2018) Global estimates of damaging hail hazard. Weather Clim Extremes 22:10–23. 10.1016/j.wace.2018.10.004

[CR60] Púčik T, Castellano C, Groenemeijer P, Kühne T, Rädler AT, Antonescu B, Faust E (2019) Large Hail incidence and its economic and societal impacts across Europe. Mon Wea Rev 147:3901–3916. 10.1175/MWR-D-19-0204.1

[CR61] Punge HJ, Kunz M (2016) Hail observations and hailstorm characteristics in Europe: a review. Atmos Res 176–177. 10.1016/j.atmosres.2016.02.012

[CR62] Punge HJ, Bedka KM, Kunz M, Reinbold A (2017) Hail frequency estimation across Europe based on a combination of overshooting top detections and the ERA-INTERIM reanalysis. Atm Res 198:34–43. 10.1016/j.atmosres.2017.07.025

[CR63] Rädler AT, Groenemeijer P, Faust E, Sausen R (2018) Detecting severe weather trends using an additive regressive Convective Hazard Model (AR-CHaMo). J Appl Meteor Climatol 57:569–587. 10.1175/JAMC-D-17-0132.1

[CR64] Rädler AT, Groenemeijer PH, Faust E et al (2019) Frequency of severe thunderstorms across Europe expected to increase in the 21st century due to rising instability. npj Clim Atmos Sci 2:30. 10.1038/s41612-019-0083-7

[CR65] Santos JA, Belo-Pereira (2019) M.A comprehensive analysis of hail events in Portugal: climatology and consistency with atmospheric circulation. Int J Climatol 39:188–205. 10.1002/joc.5794

[CR67] Sioutas M (2019) Hailfall climatology, agricultural damage and hail suppression in Greece. Le Changement Climatique, La variabilité et les risques climatiques 579. XXXIIème Colloque International de l’AIC

[CR66] Sioutas et al (2009) Hail frequency, distribution and intensity in Northern Greece. Atm Res 93:1–3. 10.1016/j.atmosres.2008.09.023

[CR68] SPC (2014) : Significant hail parameter (SHIP): [Parameter description http://www.spc.noaa.gov/exper/mesoanalysis/help/help_sigh.html.

[CR69] Stratton RA, Senior CA, Vosper SB, Folwell SS, Boutle IA, Earnshaw PD, Kendon E, Lock AP, Malcolm A, Manners J, Morcrette CJ, Short C, Stirling AJ, Taylor CM, Tucker S, Webster S, Wilkinson JM (2018) A Pan-africa convection-permitting regional climate simulation with the Met Office Unified Model: CP4-Africa. J Clim. 10.1175/JCLI-D-17-0503.1

[CR70] Taszarek M et al (2020a) Severe convective storms across Europe and the United States. Part I: climatology of lightning, large hail, severe wind, and tornadoes. J Clim 33:10239–10261. 10.1175/JCLI-D-20-0345.1

[CR71] Taszarek M et al (2020b) Severe Convective storms across Europe and the United States. Part II: ERA5 environments Associated with Lightning, large Hail, severe wind, and tornadoes. J Clim 33:10263–10286. 10.1175/JCLI-D-20-0346.1

[CR72] Torralba V, Henin R, Cantelli A, Scoccimarro E, Materia S, Manzato A, Gualdi S (2023) Modelling hail hazard over Italy with ERA5 large-scale variables. Weather Clim Extremes 39:100535. 10.1016/j.wace.2022.100535

[CR73] Trapp RJ, Marion GR, Nesbitt SW (2017) The regulation of tornado intensity by updraft width. J Atmos Sci 74:4199–4211. 10.1175/JAS-D-16-0331.1

[CR75] Tuovinen JP, Punkka AJ, Rauhala J, Hohti H, Schultz DM (2009) Climatology of severe hail in Finland: 1930–2006. Mon Wea Rev 137:2238–2249. 10.1175/2008MWR2707.1

[CR76] Vinet F (2001) Climatology of hail in France. Atmos Res 56(1–4):309–323

[CR77] Wang D, Giangrande SE, Feng Z, Hardin JC, Prein AF (2020) Updraft and downdraft core size and intensity as revealed by radar wind profilers: MCS observations and idealized model comparisons. J Geophys Research: Atmos 125(11):e2019JD031774

[CR78] Webb JDC, Elsom DM, Meaden GT (2009) Severe hailstorms in Britain and Ireland, a climatological survey and hazard assessment. Atmos Res 93:587–606

[CR79] Williams KD, Copsey D, Bodas-Salcedo A, Blockley EW, Calvert D, Comer R, Davis P, Graham T, Hewitt HT, Hill R, Hyder P, Ineson S, Johns TC, Keen A, Lee RW, Megann A, Milton SF, Rae JGL, Roberts MJ, Scaife AA, Schiemann R, Storkey D, Thorpe L, Watterson IG, Walters DN, West A, Wood R, Woollings T, Xavier PK (2018) The Met Office global coupled model 3.0 and 3.1 (GC3.0 and GC3.1) configurations. J Adv Model Earth Syst 10(2):357–380. 10.1002/2017MS001115

[CR80] Wilson DR, Ballard SP (1999) A microphysically based precipitation scheme for the UK Meteorological Office Unified Model. Q J R Meteorol Soc 125:1607–1636. 10.1002/qj.49712555707

[CR81] Wood N, Staniforth A, White A, Allen T, Diamantakis M, Gross M, Melvin T, Smith C, Vosper S, Zerroukat M, Thuburn J (2014) An inherently mass-conserving semi-implicit semi-lagrangian discretisation of the deep-atmosphere global nonhydrostatic equations. Q J R Meteorol Soc 140:1505–1520. 10.1002/qj.2235

[CR82] Ziegler CL, Ray PS, Knight NC (1983) Hail growth in an Oklahoma multicell storm. J Atmos Sci 40(7):1768–1791. 10.1175/1520-0469(1983)040%3C1768:HGIAOM%3E2.0.CO;2

